# Ischemic preconditioning modulates ROS to confer protection in liver ischemia and reperfusion

**DOI:** 10.17179/excli2017-166

**Published:** 2017-04-07

**Authors:** Phillip Bystrom, Nicole Foley, Luis Toledo-Pereyra, Kelly Quesnelle

**Affiliations:** 1 Western Michigan University, Homer Stryker M.D. School of Medicine Department of Biomedical Sciences; 2 Western Michigan University, Homer Stryker M.D. School of Medicine Department of Surgery

**Keywords:** ischemia, reperfusion injury, liver, preconditioning, reactive oxygen species, oxidative stress

## Abstract

Ischemia reperfusion (IR) injury is a significant cause of morbidity and mortality in liver transplantation. When oxygen is reintroduced to the liver graft it initiates a cascade of molecular reactions leading to the release of reactive oxygen species (ROS) and pro-inflammatory cytokines. These soluble mediators propagate a sterile immune response to cause significant tissue damage. Ischemic preconditioning (IPC) is one method that reduces hepatocellular injury by altering the immune response and inhibiting the production of ROS. Studies quantifying the effects of IPC in humans have demonstrated an improved liver enzyme panel in patients receiving grafts pretreated with IPC as compared to patients receiving the standard of care. In our review, we explore current literature in the field in order to describe the mechanism through which IPC regulates the production of ROS and improves IR injury.

## Introduction

Reactive oxygen species (ROS) and oxidant stress are the most significant pathologic mediators of ischemia reperfusion (IR) injury (Jaeschke et al., 2012[33]). It is postulated that limiting the amount of ROS produced during reperfusion would significantly benefit patients undergoing liver transplantation, liver resection, or other procedures that cause ischemia reperfusion injury. Ischemia reperfusion injury is considered a major cause of primary graft non-function following liver transplantation, and primary graft non-function is characterized by high rates of mortality (Lemasters and Thurman, 1995[45]; Ploeg et al., 1993[62]; Strasberg et al., 1994[75]). Liver transplantation ischemia is a type of cold ischemia, where the organ lacking blood flow is simultaneously cooled during ischemia. The resultant ischemia reperfusion injury is characterized by detachment of the sinusoidal endothelial cells (SECs), which may be caused by actin disassembly and activation of matrix metalloproteinases (MMPs) (Clavien et al., 1992[14]; Upadhya et al., 1997[86]; Upadhya and Strasberg, 2000[87]). Although SECs remain alive during periods of cold ischemia, they experience accelerated apoptosis during oxygenated reperfusion, likely due to the increased presence of ROS. ROS are the primary mediators of damage sustained in ischemia reperfusion injury. There are many sources of ROS production during ischemia reperfusion injury in the liver. This review will focus on the modulation of reactive oxygen species by ischemic preconditioning (IPC) as a means to ameliorate ischemia reperfusion injury.

## ROS Mediate IR Injury

Initially during IR injury, hepatocytes generate ROS from mitochondrial or cytosolic enzymes. Although hepatocellular-derived ROS do not significantly contribute to cellular injury, they stimulate the release of nuclear protein high-mobility group box 1 (HMGB1). HMGB1 is a damage-associated molecular pattern (DAMP) released from ischemic hepatocytes in response to cell damage. This soluble factor migrates out of hepatocytes and binds to TLR-4 on the surface of Kupffer cells, activating the sterile immune response and generating additional ROS. The reactive oxygen intermediates released by Kupffer cells recruit CD4+ T-cells which activate additional ROS production by Kupffer cells.

Hepatic IR injury occurs through an early and a late phase. Initially, Kupffer cells drive the early phase of hepatic injury by activating the inflammatory cascade. Kupffer cells, activated by HMGB1 in the vasculature (Yang et al., 2013[90]), generate ROS during the early stages of hepatocellular damage following IR (Jaeschke and Farhood, 1991[31]). Reactive oxygen species integral to the early phase of IR injury include hydrogen peroxide and hyperchlorous acid, both of which are taken up by hepatocytes to induce necrotic pathways of hepatocellular death (Jaeschke and Woolbright, 2012[33]). Kupffer cell activation leads to the secretion of pro-inflammatory cytokines and propagation of the sterile immune response.

The small amount of ROS generated by hepatocytes at the onset of IR injury, in conjunction with cytokine release from Kupffer cells, attracts neutrophils to the site of damage. Once inside the liver, neutrophils exert their effects during the late phase of ischemia reperfusion injury. Neutrophils are the key modulators of hepatocellular damage because of the large amount of additional ROS they produce. Neutrophil-mediated oxidant stress is first seen 6 to 24 hours following initiation of reperfusion (Hasegawa et al., 2005[27]; Jaeschke et al., 1992[30]). Neutrophils generate large amounts of myeloperoxidase, an enzyme that produces hypochlorous acid in the extracellular space (Thomas et al., 1983[83]). 

Reactive oxygen species generated by hepatocytes during ischemia reperfusion injury primarily originate from the mitochondria, although ROS generation can also occur outside of the mitochondria. Intracellular oxidant stress is generated by xanthine oxidase (XOD) (Jaeschke et al., 1988[32]), although the role of XOD-generated ROS is thought to be minute compared to mitochondria-generated ROS, which are the primary generators of intracellular ROS during IR. The mechanisms by which mitochondria produce ROS are highly variable and depend on the presence of oxygen. During the ischemic phase, blood flow restriction causes cell metabolism to shift to anaerobic respiration, therefore interrupting oxidative phosphorylation. The restriction of the electron transport chain causes a build-up of reduced electron carriers that are rapidly oxidized at the onset of reperfusion. The primary ROS mediating cellular injury is superoxide, which originates from the electron transport chain at either complex I or complex III. During the ischemic phase, superoxide production from complex III predominates (Guzy and Schumacker, 2006[25]); however, the reperfusion phase accounts for the majority of superoxide production via complex I of the electron transport chain (Murphy, 2009[55]). Superoxide production at complex I is enhanced by the elevated NADH/NAD+ ratio at the onset of reperfusion (Kussmaul and Hirst, 2006[43]). The increase in superoxide enhances formation of hydrogen peroxide and hydroxyl radicals, ultimately creating a sharp surge in ROS (Datta et al., 2013[18]; Klinman, 2007[39]). 

## IPC Reduces Mitochondrial ROS Production to Reduce IR Injury

IPC limits ROS formation and subsequently reduces ischemic injury after reperfusion (Carini et al., 2001[8]). Overall, there is a reduced inflammatory response in IPC, in addition to less oxidant stress following prolonged ischemia (Peralta et al., 2002[59]). IPC strengthens antioxidant systems inside the cell (Schauer et al., 2003[67]), induces heme oxygenase-1 (Katori et al., 2002[37]), and promotes regeneration by activating p38 and MAP kinases (Teoh et al., 2002[82]). Evidence suggests the protective effect of IPC arises from the moderate post-ischemic oxidant stress produced during preconditioning (Sindram et al., 2002[72]; Zahler et al., 2000[93]). 

Previous studies have demonstrated that even remote IPC, where ischemic preconditioning is conducted at a different organ site than the site where injury is sustained, improves perfusion and oxygenation to parenchymal tissue (Kanoria et al., 2006[36]; Tapuria et al., 2009[80]). This remote IPC is correlated with decreased hepatocellular damage in the early phase of IR injury. The main cause of cold liver ischemia reperfusion injury appears to be non-parenchymal cell injury, ultimately leading to liver graft failure in transplantation (Caldwell-Kenkel, 1989[7]; McKeown et al., 1988[52]). IPC protects against the loss of viability of SECs and the activation of Kupffer cells, both non-parenchymal types of ischemia-related, oxidant-mediated cell injury (Arai et al., 1999[2]). The loss of SECs during IR injury normally causes microcirculatory disturbances and propagates ischemia, leading to significant hepatic necrosis (Cywes et al., 1993[17]; Takei et al., 1991[79]). During remote IPC, the contralateral half of livers that did not undergo ischemia preconditioning also showed decreased non-parenchymal cell killing suggesting protection is mediated by a soluble factor. Indeed, later studies have shown that humoral factors released as a result of IPC mediate cytoprotection in the non-parenchymal cells of the liver, leading to the conclusion that whole-liver IPC produces nearly the same protection for SECs as IPC with only the half-liver (Arai et al., 2001[3]).

In addition to loss of SECs, the activation of Kupffer cells is another mechanism contributing to graft failure in liver transplantation (Caldwell-Kenkel et al., 1991[6]). During cold liver storage and subsequent reperfusion, Kupffer cells are activated and produce superoxide radicals (Caldwell-Kenkel et al., 1995[5]; Mochida et al., 1994[53]). These radicals initiate inflammatory responses by promoting neutrophil recruitment and therefore contribute to graft failure (Koo et al., 1992[40]; Lichtman and Lemasters, 1999[47]; Takei et al., 1991[79]). IPC, however, has been shown to decrease the amount of superoxide produced by individual Kupffer cells during cold liver IR (Arai et al., 2001[3]).

The prevalence of mitochondrial-derived ROS in ischemia has been demonstrated through the use of fluorescent probes. Various groups measured ROS production in cells with and without functioning mitochondria under hypoxic conditions. They discovered that only cells equipped with functional mitochondria produced a significant increase in ROS (Chandel et al., 1998[10]; Murphy, 2009[55]; Turrens, 2003[85]). This information suggests that mitochondrial-derived ROS serve as the primary source of ROS during reperfusion. 

Nitric oxide (NO) and its metabolites have been shown to protect cells following ischemia reperfusion injury, which may partially be mediated by reversible S-nitrosation of cysteines (Duranski et al., 2005[20]). This mechanism has been used to explain cellular protection in the reperfusion phase following IPC (Sun et al., 2013[77], 2007[78]). Reversible S-nitrosation occurring specifically at the exposed Cys39 in the ND3 subunit of complex I in the electron transport chain accounts for some of the cellular protection following IPC. As a gatekeeper for oxidative phosphorylation, complex I initiates the movement of electrons from NADH across the mitochondrial membrane to create a proton gradient. S-nitrosation of complex I causes mitochondria to be in a low activity conformation resulting in less ROS production (Chouchani et al., 2013[13]). By decreasing the amount of mitochondrial-derived ROS, IPC reduces oxidative damage and tissue necrosis.

## IPC Inhibits HMGB1 Release During IR

HMGB1 is a DAMP that plays a pivotal role in coordinating the sterile immune response during cold liver IR (Muller et al., 2004[54]). Hepatocytes release HMGB1 during IR in a ROS-dependent process that is mediated by post-translational acetylation. Briefly, ROS generated during IR inhibit nuclear histone deacetylases (HDACs). This results in hyperacetylation of HMGB1, which in turn results in HMGB1 translocation from the nucleus to the cytoplasm where it can be secreted into the extracellular space (Evankovich et al., 2010[21]; Yu, Tang et al., 2015[92]). 

Once in the extracellular space HMGB1 binds to various receptors on multiple target cells and plays a pivotal role in coordinating the sterile immune response (Kang et al., 2014[35]). For instance, HMGB1 signals through the NF-κB pathway in hepatocytes and stimulates the production of the proinflammatory cytokine TNF-α (Lotze and Tracey, 2005[50]). In the later stages of the sterile immune response, HMGB1 binds to TLR4 receptors on neutrophils and stimulates neutrophil extracellular trap (NET) secretion which leads to hepatocyte death and activation of Kupffer cells (Huang et al., 2015[29]). It should thus come as no surprise that inhibition of the TLR4 receptor during a sterile immune response is protective against hepatocellular injury (Mcdonald et al., 2015[51]). In sum, mitochondrial ROS generated as a result of IR stimulates the secretion of HGMB1, which in turn recruits immune cells and results in a localized sterile immune response.

**Figure 1:**
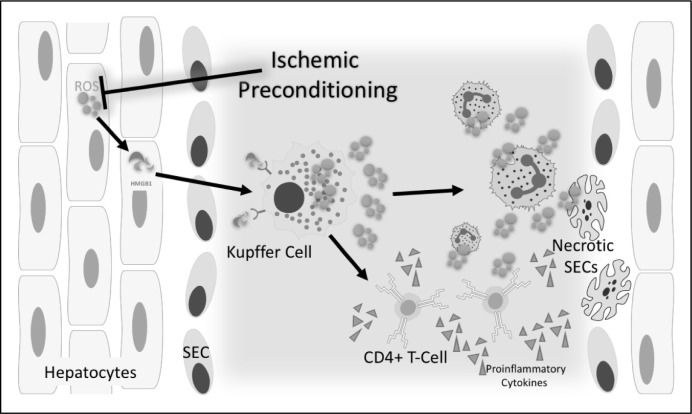
Ischemic preconditioning (IPC) inhibits hepatocyte ROS production and reduces HMGB1 release. IPC-medaited inhibition of hepatocyte reactive oxygen specied (ROS) production results in decreased levels of HMGB1 and subsequent prevention of Kupffer cell activation and downstream activation of neutrophils and CD4+ T-cells. The result is protection from immune-mediated damage to sinusoidal endothelial cells (SECs).

Since HMGB1 is central in eliciting the sterile immune response during IR, finding ways to inhibit its actions could protect against liver injury. Indeed, inhibition of HMGB1 with a neutralizing antibody was found to significantly reduce the extent of IR-induced liver damage in a murine model (Tsung et al., 2005[84]). To take this one step further, inhibition of mitochondrial ROS generation should inhibit HMGB1 secretion because secretion of HMGB1 is dependent upon intracellular levels of ROS. In fact, IR-induced HMGB1 expression in the liver was attenuated by IPC in a murine model (Garab et al., 2014[23]). In another study involving rat myocardium, IPC was also found to reduce circulating levels of HMGB1 and protect the myocardium against a sterile immune response as evidenced by a smaller area of myocardial infarct (Zhang et al., 2013[94]). In a human study on patients undergoing coronary angioplasty, IPC resulted in attenuation of the inflammatory markers CD40L, P-selectin, and myeloperoxidase (Lee et al., 2005[44]; Xu et al., 2016[89]). These markers are upregulated in the presence of HMGB1 and are involved in immune activation and thus contribute to the sterile response seen in IR. Taken together, these studies demonstrate the ability of IPC to inhibit HMGB1 release and subsequent immune activation during preclinical models of IR (Figure 1(Fig. 1)). 

## IPC Inhibits Expression of NF-κB and Proinflammatory Cytokines During IR

**Figure 2:**
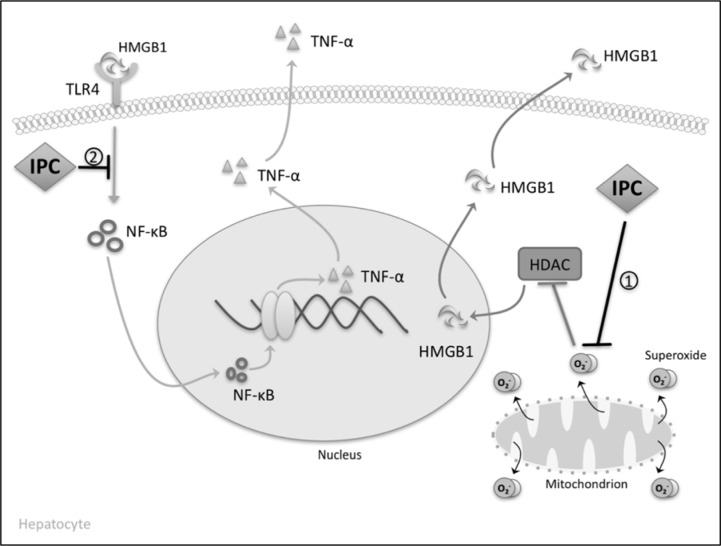
In the absence of IPC, reactive oxygen species such as superoxide inhibit HDACs causing hyperacetylation of HMGB1 in the nucleus. In this form HMBG1 translocates to the cytoplasm and eventually enters the extracellular space to coordinate the sterile immune response. (1) IPC exerts its cytoprotective effects by decreasing the amount of superoxide produced intracellularly, thereby disinhibiting HDACs from acting on HMGB1 in the nucleus. HMGB1 is then unable to move into the extracellular space to drive immune activation. (2) The decreased amount of HMGB1 in the vasculature reduces signaling through the NF-kB pathway, thereby suppressing the production of proinflammatory cytokines such as TNF-alpha.

NF-κB is a transcription factor that plays a central role in cytokine production and cell survival (Gilmore, 2006[24]). NF-κB is upregulated in hepatocytes during IR and stimulates the release of several proinflammatory cytokines that contribute to the sterile immune response (Ricciardi et al., 2000[64]; Shin et al., 2008[70]). Notably, the proinflammatory cytokine TNF-α plays a central role in the sterile immune response and is responsible for a significant portion of the liver injury associated with IR (Perry et al., 2011[61]). NF-κB expression is enhanced during IR by several mechanisms: HMGB1 signaling via cell surface receptors (Park et al., 2004[57]), increased intercellular Ca^2+^ concentrations during ischemia that results in the activation of protein kinase C which in turn activates NF-κB (Steffan et al., 1995[74]), stimulation of NADPH oxidase with subsequent ROS production during ischemia (Spencer et al., 2013[73]), and accumulation of mitochondria-derived ROS during IR (Chandel et al., 2000[11]) (Figure 2(Fig. 2)).

Once activated, NF-κB translocates to the nucleus where it acts as a transcription factor that stimulates the production of numerous proinflammatory molecules. Since NF-κB plays such a pivotal role in the inflammatory response, inhibiting its actions prevents against tissue damage during IR. Inhibition of NF-κB in mice with the small molecule inhibitor Baicalein provided considerable protection against IR injury of the liver (Liu et al., 2015[49]). Several studies indicate that IPC inhibits NF-κB during IR and thus inhibits the production of proinflammatory cytokines. In a murine model, IPC attenuated NF-κB activity in hepatocytes and led to a reduction in TNF-α and ICAM-1 mRNA levels when subject to subsequent IR. IPC also inhibited phosphorylation of the NF-κB inhibitor (IκB), thereby prolonging its half-life and its ability to inhibit NF-κB. Together, these findings suggest that IPC inhibits NF-κB activity and the subsequent production of proinflammatory cytokines (Funaki et al., 2002[22]). 

In a separate study involving rat myocardium, IPC led to a similar inhibition of NF-κB activity with a corresponding reduction in levels of proinflammatory cytokine mRNA (IL-1, IL-6, TNF-α). IPC was also found to protect the myocardium from IR-induced tissue injury as evidenced by diminished infarct size as compared to a control group that did not undergo IPC. The authors conclude that inhibition of NF-κB via IPC attenuates proinflammatory cytokine production, which protects the myocardium from IR injury (Hiasa et al., 2001[28]). A third study involving a murine model similarly found that IPC inhibited NF-κB activity, thereby attenuating TNF-α expression upon exposure to lipopolysaccharide. Mice were randomly assigned to be either controls or to undergo remote IPC in which blood flow to a hind limb was occluded with a rubber band three times for ten minutes each time and separated by a ten minute period of reperfusion. Remote IPC was found to inhibit NF-κB activity with concomitant attenuation of TNF-α expression, neutrophil accumulation, and microabscess formation in the liver, thus protecting the liver from subsequent IR insult (Shin et al., 2014[69]). Finally, IPC was shown to attenuate expression of TNF-α and ICAM-1 in hepatocytes using a murine model of IR. Mice assigned to undergo IPC had significantly reduced levels of circulating ALT and LDH than did the mice assigned to the sham-operated group. IPC was also found to inhibit neutrophil infiltration and the authors attribute these findings to suppression of NF-κB via IPC (Li et al., 2006[46]). Taken together, these studies suggest that IPC inhibits the activity of NF-κB, resulting in a reduction of proinflammatory cytokine production, which protects the liver during IR. 

## IPC Inhibits Kupffer Cell Activation During Cold IR

Kupffer cells are tissue-specific macrophages that line the sinuses of the liver and serve as the primary source of ROS during the early stages of the sterile immune response. The ROS and proinflammatory cytokines generated by Kupffer cells lead to the recruitment of neutrophils which then serve as the primary source of ROS during the later stages of the sterile immune response (Jaeschke and Farhood, 1991[31]). Kupffer cells are activated by the HMGB1 that is secreted by hepatocytes in response to IR (Yang et al., 2013[90]). HMGB1 signals through Kupffer cell TLR4 receptors to stimulate the production of ROS and other proinflammatory molecules that lead to widespread tissue injury (Kupiec-Weglinski and Busuttil, 2005[42]; Tsung et al., 2005[84]). Inhibition of Kupffer cell activation during IR may suppress inflammation and thus protect the liver from injury. In two separate studies involving preclinical animal models, IPC inhibited Kupffer cell activation and protected against liver tissue injury. In these studies, rats were separated into two groups and underwent either IPC or sham surgery. IPC involved clamping of the hepatic artery and portal vein for five minutes followed by five minutes of reperfusion. Upon subsequent IR insult, Kupffer cell activity was suppressed in the group that underwent IPC as evidenced by diminished superoxide production in the vicinity of the Kuppfer cells. In addition, IPC provided significant protection to liver tissue. The authors measured the extent of tissue injury by counting the number of nonviable SECs following IR insult and found that IPC reduced SEC death by more than half. The authors attribute this tissue preservation to Kupffer cell inactivation during IPC (Arai et al., 1999[2], 2001[3]). It is interesting to note that whereas the protection conferred by IPC during cold IR is thought to be the result of decreased Kupffer cell activity, the opposite is believed to be true regarding warm IR. That is the cytoprotection conferred by IPC during warm IR insult is thought to be the result of an increase in ROS. This sublethal burst of Kuppfer cell-derived ROS is thought to prime the organ for subsequent IR insult. Indeed, Tejima et al. (2004[81]) showed that there was an increase in Kupffer cell derived ROS during warm IPC. Furthermore, they were able to mimic the cytoprotective effects of IPC by pretreating rat livers with low concentrations of hydrogen peroxide. In either case, it is clear that the Kuppfer cells play a central role in the cytoprotective properties of IPC.

In another study, IPC of rat livers was found to inhibit TNF-α secretion from Kupffer cells during IR in a process mediated by NO. IPC suppressed inflammation and protected the lungs and the liver from tissue injury. The authors showed that Kupffer cell inhibition with gadolinium chloride inhibited secretion of TNF-α during IR and attenuated the harmful inflammatory response. When TNF-α was reintroduced into the tissue, the anti-inflammatory effects of gadolinium chloride were reversed. These findings suggest that Kupffer cells are the primary source of TNF-α immediately following IR. The authors then show that IPC provides similar inhibition of TNF-α and suppression of the inflammatory response seen with gadolinium chloride. This suggests that IPC protects against tissue injury by inhibiting the secretion of TNF-α from Kupffer cells. Finally, the authors showed that the protective effects conferred by IPC are lost when NO synthesis is inhibited, which indicates that the process is mediated by NO. The authors conclude that IPC inhibits TNF-α secretion by Kupffer cells in a NO-dependent process. This supresses a harmful inflammatory response induced by IR and protects the liver and lungs against injury (Peralta et al., 1999[60]). Taken together, these findings seem to indicate that IPC inhibits Kupffer cell activity and in doing so attenuates IR-induced inflammation. 

## IPC Inhibits CD4+ T-Cell Recruitment and Activation During IR

Activated Kupffer cells secrete ROS and proinflammatory cytokines such as IL-6 and TNF-α that recruit CD4^+^ T-cells to the the liver during IR. Kupffer cell cytokines also upregulate T-cell specific adhesion molecules on SECs to facilitate T-cell recruitment to the site of insult (Jaeschke and Farhood, 1991[31]). Once in the liver, activated CD4^+^ T-cells can reciprocally activate Kupffer cells in a CD-40-dependent process thus leading to a self-perpetuating inflammatory response (Hanschen et al., 2008[26]). CD4^+^ T-cells are responsible for significant tissue injury during IR and inhibition of CD4^+^ T-cell activation could have therapeutic potential (Shen et al., 2009[68]). 

Several studies have implicated IPC in CD4^+^ T-cell inhibition during IR. In one study, mice were divided into two groups. One group underwent 30 minutes of renal IPC while the other group underwent a sham operation. Five days after the operation, leukocytes were purified from the spleens of each group and injected into the circulation of T-cell deficient mice. Upon exposure to IR insult, the mice that received leukocytes from IPC mice showed markedly less kidney injury than the mice that received leukocytes from the sham-operated group. This study suggests that IPC inhibits the capacity of leukocytes to cause tissue injury during subsequent bouts of IR. Although the authors did not elucidate the specific populations of leukocytes that were affected by IPC, they were able to rule out neutrophils and macrophages since their infiltration into the kidney did not provide any tissue protection (Burne-Taney et al., 2006[4]). 

A second study in a murine kidney model found that short bursts of IPC resulted in a significant reduction in the activity of the stress-activated mitogen activated protein (MAP) kinases JNK and p38, which was accounted for by inactivation of upstream MAP kinase kinases. JNK and p38 have long been known to serve as growth and survival factors and thus it is somewhat counterintuitive that a reduction in these mediators is actually cytoprotective during IR. Briefly, IR insult causes oxidative stress that stimulates increased expression of the stress-activated mediators JNK and p38. These mediators in turn stimulate expression of specific adhesion molecules on the cell surface that assist in the recruitment of leukocytes to the site of injury (Read et al., 1997[63]). Once recruited to the target site, the leukocytes propagate the sterile immune response by secreting proinflammatory mediators that lead to inflammation and subsequent tissue damage (Xia et al., 1995[88]). The authors of the study argue that inhibition of this pathway resulted in diminished cytokine-induced leukocyte-endothelial interactions, which prevented recruitment of leukocytes to the site of insult, thus preventing leukocyte-induced tissue damage (Park et al., 2001[58]). IPC thus appears to inhibit leukocyte recruitment during IR. 

In a third study, human subjects undergoing elective cruciate ligament surgery were divided into a group that received IPC and a group that did not. The group that underwent IPC was subject to three five-minute rounds of tourniquet-induced ischemia separated by a five-minute period of reperfusion. Both groups were then subject to one hour of tourniquet-induced ischemia during surgery. After surgery, the group that underwent IPC exhibited marked inhibition of proinflammatory cytokine production by CD4^+^ T-cells and thus attenuated an inflammatory immune response. In particular, circulating levels of the proinflammatory cytokine IL-2 produced by CD4^+^ T-cells was significantly reduced in the group that underwent IPC (Sullivan et al., 2009[76]). 

IPC also affects the activity of immunosuppressing regulatory T cells (T_regs_). In two separate studies involving murine models of renal IPC, IPC activated T_regs_ which reduced immune-mediated inflammation and protected against tissue injury. In particular, T_regs_ harvested from mice that underwent either IPC or sham surgery were injected into naïve mice. Those animals that received T_regs_ from donor mice that underwent IPC exhibited similar tissue protection and suppression of inflammation, indicating that IPC-activated T_regs_ protect against subsequent IR insult. (Cho et al., 2010[12]; Kinsey et al., 2010[38]). Taken together, this data suggests that IPC negatively regulates the immune response during IR and thus protects against tissue damage.

## IPC Improves Liver Function Following IR Injury in Humans

In humans, the efficacy of IPC can be measured several ways post-transplantation. Quantifiable assays to measure the degree of ischemic injury in the liver include serum bilirubin, aspartate transaminase (AST), and alanine aminotransferase (ALT). Other measures such as lactic acid are also used to measure liver function indirectly. Lactic acid is a product of cell metabolism and can accumulate in states of hypoxia. In the context of liver transplantation, serum lactate levels will increase in conjunction with the severity of hypoxia. Normalization of blood lactate levels can indicate recovery of liver metabolic capacities in the postoperative setting of liver transplantation (De Gasperi et al., 1997[19]). 

In studies of IPC in human liver transplantation, AST and ALT levels are used to indicate protective effects due to IPC. Transaminase levels following liver transplantation are the most reliable markers of hepatocellular injury. Of the cellular markers, serum AST is most tightly correlated with the degree of ischemic injury to the liver (Clavien et al., 2000[15]). The reduction of AST levels in the patient is associated with a reduction in cell death during the late preconditioning phase of IPC (Jassem et al., 2009[34]). IPC has been shown to decrease levels of circulating ALT, AST, and LDH (Koti et al., 2003[41]; Liu et al., 2014[48]; Ricciardi et al., 2001[65]; Sindram et al., 2002[72]; Zhu et al., 2014[95]).

In human liver transplantation preceded by IPC, postoperative AST and ALT levels were significantly lower than that of the control group (Clavien et al., 2003[16]). When used with allografts of deceased donor liver for transplantation, IPC protects the organ by reducing AST levels in the recipient. Three days after transplant, AST levels were lower in patients who received an allograft treated with IPC prior to transplantation than those who received an allograft that was not preconditioned. Patients that received a liver allograft pretreated with IPC also demonstrated lower serum lactate levels compared to untreated one day post-transplantation (Jassem et al., 2009[34]).

## Limitations of IPC in the Clinical Setting

Although IPC initially leads to a favorable reduction in numerous biomarkers for liver damage in the early stages of IR, in the long term it does not appear to have any effect on overall morbidity and mortality following liver resection or transplantation (Zhu et al., 2014[95]). During liver resection circulation to the organ is temporarily interrupted which increases the risk for IR injury when blood flow is restored. A randomized control trial involving 84 patients that underwent major liver resection found no clinical benefit to IPC prior to resection using an intermittent Pringle maneuver (Scatton et al., 2011[66]). In a similar study involving 100 patients, IPC prior to liver resection also using a Pringle maneuver did not provide any clinical benefit in terms of length of hospitalization, morbidity, or mortality (Ye et al., 2014[91]). A study involving 22 participants undergoing adult-to-adult living liver transplantation did not find any clinically significant benefit for the donor nor the recipient in terms of morbidity or mortality (Andreani et al., 2010[1]). Although another trial involving 47 whole liver transplantations from deceased donors found that IPC prevented hepatocellular necrosis, this did not appear to provide any clinically significant benefit for the patient (Cescon et al., 2006[9]). A metaanalysis of 11 randomized control trials involving 669 patients that underwent liver resection also failed to find any statistically significant difference in morbidity or mortality rates among study participants (O'Neill et al., 2013[ref:56]). Thus, although it is well documented that IPC provides early protection against IR by attenuating mitochondrial ROS production and thus sparing hepatocytes in the early stages of IR injury, there does not appear to be any convincing evidence that IPC provides any clinically demonstrable benefit in reducing patient morbidity or mortality following liver resection or transplantation.

It has been suggested that the discrepancy between preclinical and human modeling of IPC efficacy may be due to variable timing and type of vascular occlusion in IPC protocols in human trials, as well as the number of cirrhotic and steatotic livers included in the human trials. Importantly, although changes in mortality are not observed in the human trials performed to date, other beneficial positive outcomes such as reduced operative blood loss are improved in the setting of IPC (Simillis et al., 2016[71]). Taken together, these data identify a need for further randomized clinical trials to compare length of ischemia during preconditioning, methods of vascular occlusion, and subtypes of patients who may benefit most from IPC.

## Conclusion

IR injury is a significant source of morbidity and mortality following liver transplantation. When circulation is restored to a newly transplanted liver, the reintroduction of oxygen into the tissue sets off a sterile immune response that can result in widespread tissue injury and transplant rejection. IPC has emerged as a means to mitigate such tissue damage and its ability to attenuate IR-induced tissue injury in the laboratory has been well documented in the literature. However, although IPC leads to a favorable reduction in biomarkers for tissue injury in the laboratory, these findings have not been successfully translated into clinical practice, as there is limited evidence that IPC has an influence on post-transplantation morbidity and mortality. Future work should be aimed at finding a way to translate these promising laboratory findings into clinical practice.

## Notes

Phillip Bystrom and Nicole Foley contributed equally to this work.

## Conflict of interest

The authors declare that they have no conflict of interest.
